# Pulmonary sarcoidosis masquerading as metastatic cervical cancer: a pitfall in CT scans and positron emission tomography (PET)

**DOI:** 10.1259/bjrcr.20190125

**Published:** 2020-05-14

**Authors:** Jeffrey Rubasingham, Sherief Marzouk, Krishnaswamy Madhavan, George Sioftanos, Sidath H Liyanage

**Affiliations:** 1 Southend University Hospital NHS Foundation Trust, England, United Kingdom

## Abstract

Sarcoidosis has been associated with co-existing malignancies in several organs, including the breast and thyroid gland as well as lymphomas. However, the occurrence of sarcoidosis with cervical cancer is rare with only nine previous cases reported in the published literature. We present a case of pulmonary sarcoidosis imitating mediastinal lymph node metastases on the staging CT scan and positron emission tomography imaging. The presence of thoracic lymphadenopathy without any pelvic lymphadenopathy prompted histological confirmation of sarcoidosis on endobronchial ultrasound guided biopsy. Misdiagnosing pulmonary sarcoidosis as metastases would have precluded the patient from receiving the curative treatment and likely resulted in suboptimal outcomes.

## Clinical presentation

A 31-year-old female presented with a history of post-coital bleeding. She had never had a cervical smear or any other form of screening and did not have any significant past medical history. She had completed her family with two children, worked as a lorry loader and smoked occasionally. There was no shortness of breath, cough or any other respiratory symptoms. A thorough systemic inquiry did not reveal any other significant symptoms. On examination, there was a large fungating polyp on the cervix. Respiratory examination was within normal limits. There were no extra pulmonary manifestations of sarcoidosis.

## Investigations

The patient underwent a punch biopsy which showed a moderately differentiated squamous cell carcinoma of the cervix. Routine blood tests including a full blood count, urea and electrolytes, liver function tests and C-reactive protein (CRP) were all within normal limits.

**Figure 1.**
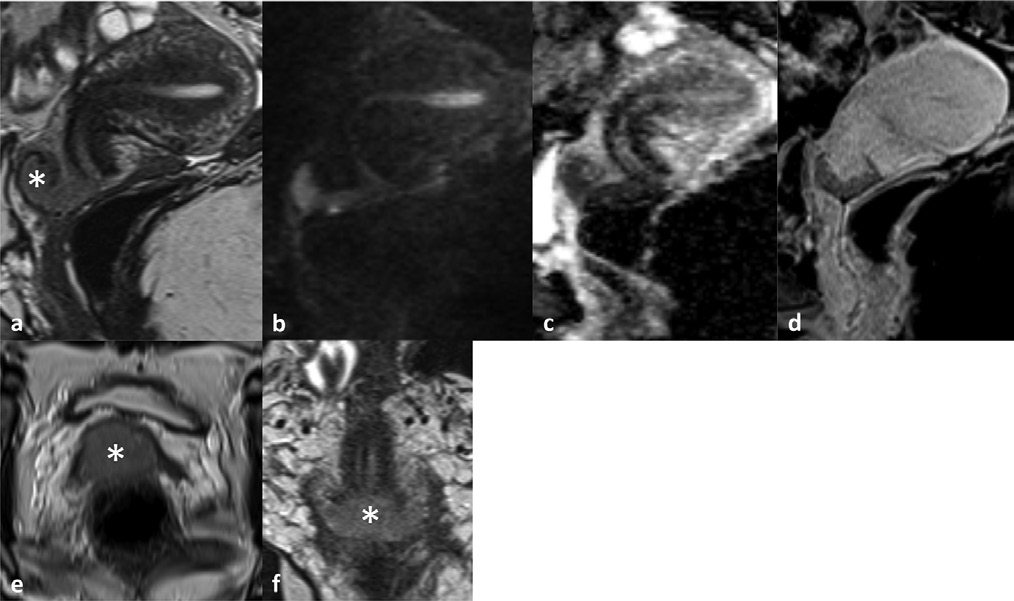
Sagittal (a), axial (e) and coronal oblique (f) T2 images showing a intermediate signal intensity (SI) mass (asterisk) in the anterior lip of the cervix which is high SI on the DWI b-800 images (b) and corresponding low SI on the ADC map (c). The mass shows hypoenhancement relative to the rest of the cervix and myometrium on the delayed phase post-contrast scans (d). ADC, apparent diffusion coefficient; DWI, diffusion-weighted imaging; SI, signal intensity.

**Figure 2.**
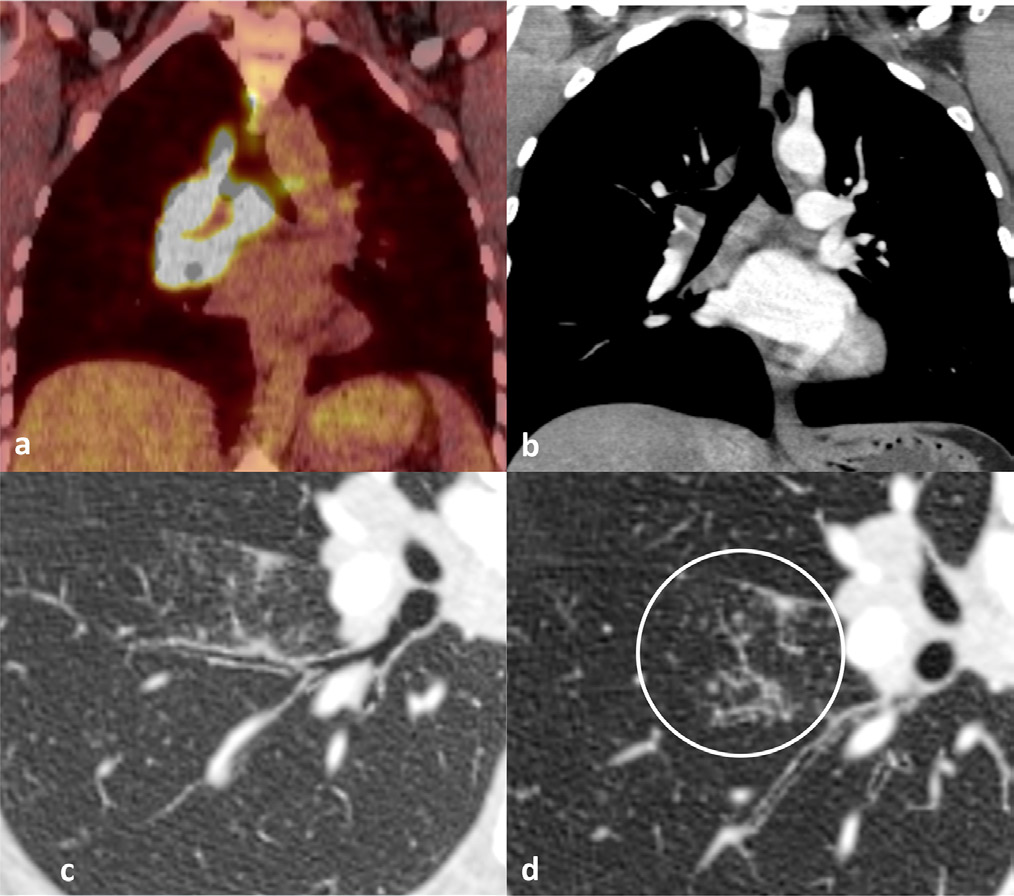
CoronalFDG-PET/CT (a) and equivalent post contrast coronalreformatted CT (b) showing right paratracheal, hilar and subcarinal lympadenopathy. The HRCT component of the CT shows asubtle focal area of thickening of the bronchovascular tree and oblique fissure (c) and tinynodules (circle) in the right lower lobe (d).

MRI of her pelvis confirmed a mass in the cervix, extending to its extreme lateral margins with a suspicion of bilateral parametrial extension, amounting to at least FIGO Stage IIB^1^ (Figure 1). In addition to this finding, a staging CT scan of the chest, abdomen and pelvis revealed a large mediastinal lymph node, right hilar and subcarinal nodes (Figure 2). There was a subtle focal area of thickening along the oblique fissure and bronchovascular tree and tiny nodules in the right lower lobe of the lung (Figure 2). Apart from small inguinal lymph nodes, no other pathological lymph nodes or visceral metastases were noted elsewhere.

**Figure 3.**
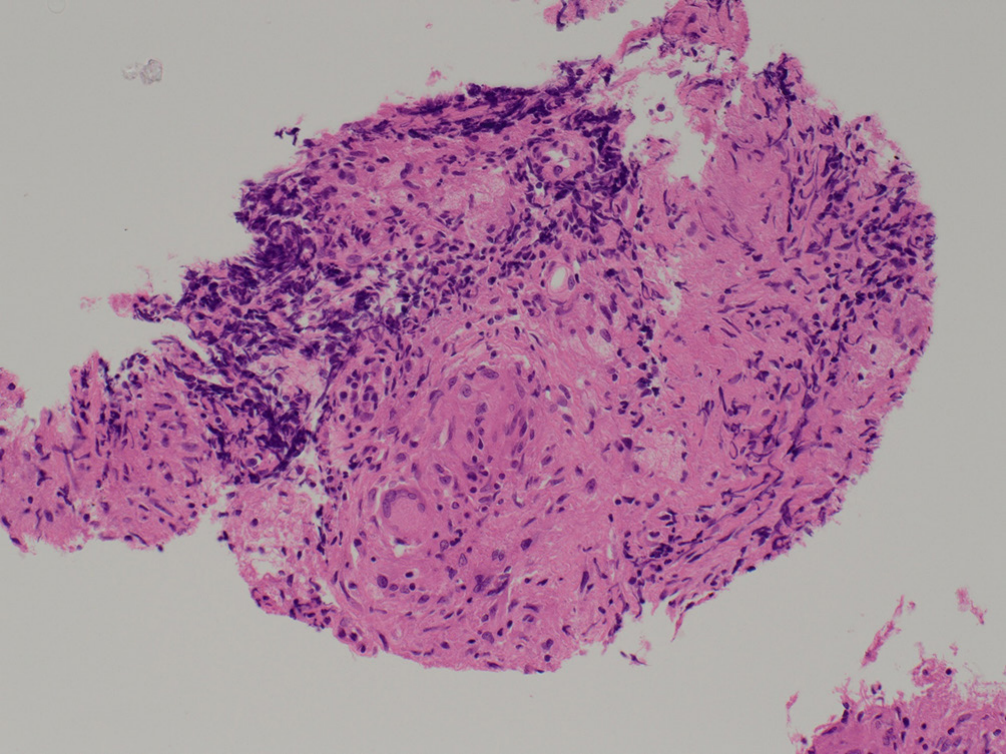
Haemotoxylin and Eosin stained section of the mediastinal lymph node showing well-formed granulomas of epithelioid cells with multinucleated giant cells and lymphocytic cuffs. Image courtesy of Dr Filomena Medeiros.

It was felt that the pattern of distribution of disease, with mediastinal lymphadenopathy and uninvolved pelvic or para-aortic nodes, was unusual for cervical cancer and the patient subsequently underwent a PET-CT (Figure 2). The PET-CT also showed 2-[^18^F]-flu-2deoxy-D-glucose (FDG) avid mediastinal and right hilar lymphadenopathy and was therefore unhelpful in differentiating metastatic disease from the other differentials discussed below. Consequently, the patient underwent an endobronchial ultrasound (EBUS) guided biopsy of the mediastinal lymph nodes. Histopathological assessment of the EBUS biopsy showed high cellularity with numerous well-formed granulomas of epithelioid cells with several multinucleated giant cells and lymphocytic cuffs. There was focal necrosis of non-caseating type.(Figure 3) The overall impression was one of pulmonary sarcoidosis.

## Differential diagnosis

At first glance, the most obvious diagnosis would appear to be mediastinal and thoracic lymphadenopathy secondary to metastatic cervical cancer. However, thoracic lymphadenopathy in the absence of pelvic and para-aortic lymphadenopathy is unusual in the context of cervical cancer. Other differentials include a second primary cancer, such as breast cancer, lymphoma, oesophageal cancer and lung cancer, especially, in view of the smoking history.^2^ Malignancies originating from these thoracic organs have different lymphatic drainage pathways and the pattern of lymphadenopathy can aid in differentiating them from each other.^3^ Non-malignant differentials for mediastinal lymphadenopathy would include inflammatory causes such as sarcoidosis, as seen in this case and also infective causes such as tuberculosis, histoplasmosis and HIV. The most common finding in sarcoidosis is bilateral hilar lymphadenopathy with right paratracheal lymphadenopathy. Parenchymal infiltration is seen in the earlier stages of disease and pulmonary fibrosis in the later stages.^4^ Tuberculosis usually presents with unilateral compromise and hilar lymph nodes are most commonly affected.^4^ Typically, there are hypodense centres due to caseous necrosis and a peripheral denser area, which increases with contrast, due to the cuff of granulomatous inflammatory tissue.^4^ Calcifications may be seen in lymphadenopathy associated with acute fungal infections.^4^

Histopathological assessment with special stains for fungi and tuberculosis did not yield positive results in the case presented. Other rarer causes of enlarged thoracic lymph nodes on CT scan include lymphoid hyperplastic conditions such as Castleman’s disease as well as idiopathic pulmonary fibrosis (IPF).^2^ Castleman’s disease is usually associated with a solitary, large, non-invasive, homogeneous mass in the mediastinum or hila, with a soft tissue attenuation.^4^ CT findings in IPF include mediastinal lymphadenopathy and associated interstitial changes.^4^ Anomalous or aberrant mediastinal blood vessels can also occasionally imitate mediastinal lymph nodes on CT scans.^2^

## Treatment

In the absence of distant metastases, the patient was staged as FIGO IIB and underwent radical treatment with concurrent chemoradiotherapy with curative intent. The treatment involved external beam radiotherapy to the pelvis with concurrent Cisplatin followed by two fractions of brachytherapy to the cervix.

## Follow-up

MRI of the pelvis, 3 months after completion of chemoradiotherapy and brachytherapy, showed complete response with no residual tumour. CT scan of the chest revealed no significant changes to the pre-existing lymphadenopathy.

## Discussion

Worldwide, cervical cancer is the fourth most common cancer amongst females and globally has a high mortality rate, with 90% of cervical cancer-related deaths occurring in low- and middle-income countries.^5^ However, in the UK, cervical cancer accounts for less than 1% of all cancer deaths amongst females and mortality rates have fallen by 74% since 1970’s,^5^ owing not only to the introduction of a screening programme, but also to the highly effective treatment options available today. The clinical management of cervical cancer is largely dependent on the stage of disease. Patients with FIGO stages I to III and even some with stage IVA disease are offered radical treatment options with curative intent whereas those with distant metastases (FIGO Stage IVB) are offered treatment with palliative intent.^6^ Thus, accurate radiological staging of cervical cancer, particularly in reporting distant metastases, is critical as it can often be the deciding factor between offering treatment with curative and palliative intent.

In the case presented here, it was the unusual pattern of distribution of presumed lymph node spread which prompted further investigations into its aetiology. Had the possibility of a non-metastatic aetiology for the thoracic lymphadenopathy not been entertained, the patient would have been inaccurately staged as FIGO Stage IVB and consequently would not have been considered for treatment with curative intent. In a 31-year-old patient with no co-morbidities and good performance status, this would have very likely resulted in an unacceptably suboptimal clinical outcome.

Sarcoidosis, a multisystem, granulomatous, inflammatory disorder with unknown aetiology has been associated with an increased risk of developing malignancies in several organs, in particular lung, liver, gastric cancers as well as melanomas and lymphomas.^7^ A previously published review identified 59 reported cases of cancer and co-existing sarcoidosis.^7^ The most common primary cancer was of breast origin (*n* = 12), followed by thyroid cancers (*n* = 8).^7^ The review identified only 1 case of cervical cancer co-existing with sarcoidosis.^7,8^ A literature search on PubMed database revealed further studies reporting co-existing sarcoidosis with cervical cancer, as summarised in Table 1.

**Table 1.** 

Author, year	Type of study	Histological type of tumour	Number of patients	Age	Manifestation of sarcoidosis
Alliot et al, 2001^9^	Case report	Squamous cell carcinoma	1	46	Multisystem sarcoidosis
Goldstein et al, 2008^10^	Case report	Squamous cell carcinoma	1	49	Mediastinal lymphadenopathy
Mapelli et al, 2013^11^	Case report	Adenosquamous carcinoma	1	26	Subcutaneous nodules and thoracic lymphadenopathy
Blank et al, 2014^12^	Retrospective observational study	Not specified	5	Not specified	Not specified
El Hammoumi et al, 2015^8^	Case report	Epidermoid carcinoma	1	47	Mediastinal and hilar lymphadenopathy

A thorough evaluation of suspected sarcoidosis warrants detailed history including occupational and environmental exposure as well as a full physical examination, focussing on the pulmonary as well as the extra pulmonary manifestations of sarcoidosis.^13^ As there is no definitive diagnostic test for sarcoidosis and it is often a diagnosis of exclusion, further tests are required to exclude other differentials such as tuberculosis, HIV and fungal infections.^13^ A definitive diagnosis of sarcoidosis requires clinical or radiological manifestations, exclusion of other differentials and histopathological confirmation of non-caseating granulomas.^13^ Differentiating sarcoidosis from other causes is difficult based on CT alone. Symmetry of hilar lymphadenopathy can be a helpful feature in sarcoidosis, differentiating it from lymphoma, TB and fungal infections.^4^ In patients with known malignancy PET-CT could be considered. Most of the studies reviewed in Table 1 reported hypermetabolic activity of the thoracic lymphadenopathy on PET imaging.^8,10,11^ FDG PET imaging is a commonly used modality in a variety of malignancies and often helps with accurate clinical staging of the disease.^11^ However, as both malignant lesions as well as inflammatory lesions such as sarcoidosis lesions engage in glucose consumption, they can both show FDG avidity and could be indistinguishable on PET imaging.^11^

## Learning points

1. Consider histopathological confirmation when there is diagnostic uncertainty on imaging, especially if the distribution of lymphadenopathy is not in keeping with the known pattern of lymph node spread for the primary malignancy.
2. Hypermetabolic activity from sarcoidosis on PET imaging is often indistinguishable from malignancy. Misdiagnosing pulmonary sarcoidosis as metastatic disease is a potential pitfall and can result in the patient not receiving the curative treatment and lead to suboptimal outcomes.
3. Despite the rare occurrence of co-existing sarcoidosis with cervical cancer, it is an important differential diagnosis to consider in cervical cancer patients with thoracic lymphadenopathy, especially in those who are otherwise suitable for curative treatment.
